# The hepatic senescence-associated secretory phenotype promotes hepatocarcinogenesis through Bcl3-dependent activation of macrophages

**DOI:** 10.1186/s13578-021-00683-5

**Published:** 2021-09-16

**Authors:** Yihua Huang, Xue Yang, Yan Meng, Changchun Shao, Jianping Liao, Fengwei Li, Rong Li, Yingying Jing, Aimin Huang

**Affiliations:** 1 Department of Pathology, School of Basic Medical Sciences, Fujian Medical University, 88 Jiaotong Road, Fuzhou, Fujian 350004 People’s Republic of China; 2 Tumor Immunology and Gene Therapy Center, Third Affiliated Hospital of Second Military Medical University, Shanghai, 200438 China; 3 Institute of Translational Medicine, Shanghai University, Shanghai, 200444 China; 4 Department of Hepatic Surgery IV, Eastern Hepatobiliary Surgery Hospital, The Second Military Medical University, Shanghai, 200438 China

**Keywords:** Liver cancer, Hepatic senescence-associated secretory phenotype, Bcl3, Macrophage

## Abstract

**Background:**

Liver cancer is one of the most common malignancies in the world with a poor prognosis. Hepatocellular carcinoma (HCC) is the most prevalent primary liver cancer, accounting for 80–90% of cases. The initiation and progression of HCC are closely associated with chronic liver inflammation. In addition, HCC is often accompanied by cell senescence. Senescent hepatocytes can secrete various inflammatory factors, collectively called the senescence-associated secretory phenotype (SASP). The SASP has been confirmed to promote the occurrence of liver cancer by affecting the inflammatory microenvironment. However, its role and the underlying mechanism of hepatic SASP in hepatocarcinogenesis are not clearly understood. Therefore, a better understanding of the pathogenic mechanisms of the effect of the hepatic SASP on the occurrence of HCC is still needed.

**Methods:**

The study aims to explore the role of SASP factors and the underlying mechanism in tumorigenesis and the progression of HCC in vivo. We used diethylnitrosamine (DEN) combined with carbon tetrachloride (CCl_4_) (DEN-CCl_4_) to establish liver cancer model in wild-type (*WT*) mice and Bcl3 knockout (*Bcl3*^*−/−*^) mice. β-galactosidase (β-gal) staining was performed to evaluate the degree of cellular senescence. Immunohistochemistry (IHC) were used to detect the degree of cellular senescence and the activation of macrophage. PCR chip and clinical tissue chip assays were used to estimate the RNA levels of SASP factors and NF-κB related genes, and their protein levels were examined by Western blot assays.

**Results:**

DEN-CCl_4_ induced cellular senescence in mouse hepatocytes. In addition, senescent hepatocytes might release a variety of inflammatory factors that further activate macrophages, thereby changing the microenvironmental state and promoting the occurrence of HCC. Mechanistically, the NF-κB pathway is important because it regulates the SASP. Therefore, we used a PCR chip to detect the expression of NF-κB-related genes in senescent liver tissue. Our results showed that the expression of Bcl3 was increased in senescent hepatocytes, and knocking out Bcl3 significantly inhibited the secretion of hepatocyte SASP factors and the activation of macrophages, thereby inhibiting hepatocarcinogenesis. Finally, in clinical tissues adjacent to HCC tissues in patients, the expression of Bcl3 and IL-8 correlated with poor prognosis in HCC patients.

**Conclusion:**

The hepatic SASP can further induce the activation of macrophages during hepatocarcinogenesis, thereby promoting the occurrence of HCC, and that this process is closely related to the expression of Bcl3 in hepatocytes.

**Supplementary Information:**

The online version contains supplementary material available at 10.1186/s13578-021-00683-5.

## Introduction

Liver cancer is the sixth most commonly diagnosed cancer and the third leading cause of cancer-related death worldwide, and it is mainly categorized into primary liver cancer and metastatic liver cancer [1]. Hepatocellular carcinoma (HCC) is the most prevalent primary liver cancer [2]. China has one of the highest incidences of HCC in the world, and HCC poses a serious threat to the health of human beings [3]. However, due to aggressive growth and late symptom presentation, most patients with HCC are diagnosed at advanced stages and are not eligible for surgical treatments [4]. Therefore, it is essential to better understand the pathogenesis of liver cancer and to identify more effective therapeutic strategies.

It is well known that chronic inflammatory reactions have been highly implicated in the initiation and development of cancer, including HCC [5]. However, the exact role and underlying mechanism of the inflammatory microenvironment in hepatocarcinogenesis remain controversial. Early studies aimed at the infiltration of inflammatory cells such as monocytes, macrophages, neutrophils, and eosinophils and showed that these cells exert direct effects on tumorigenesis. While, hepatocytes are the majority of the hepatic cells in liver. Few studies were focused on the contribution of hepatocytes to inflammatory microenvironment and their role in hepatocarcinogenesis [6].

In response to endogenous and exogenous stress, hepatocytes might enter into a state of cell cycle arrest. These cells are called senescent hepatocytes. Senescent hepatocytes can secrete a variety of factors, including proinflammatory cytokines, chemokines, growth factors, and proteases, collectively referred to as the SASP [6–8]. SASP factors secreted by senescent cells are highly dynamic, which further causes changes in the microenvironment and homeostasis and might determine their beneficial or harmful effects [6, 9, 10]. For example, some SASP factors might inhibit tumorigenesis by reinforcing growth arrest during early tumorigenesis [11–14]. However, in other cases, highly dynamic SASP factors might promote tumor progression by remodeling the tumor microenvironment [8, 12, 15, 16]. However, the detailed mechanisms underlying this process remain unclear.

Furthermore, hepatic SASP factors can recruit and activate immune cells. Activated immunocytes have tumor-promoting and tumor-suppressing activities, depending on the context [11, 12, 17–19]. Activated immune cells, including macrophages, might contribute to the elimination of precancerous senescent hepatocytes, inducing their immune-mediated clearance to prevent tumor initiation, a process termed ‘‘senescence surveillance’’ [11, 17]. Additionally, the SASP factors might establish an pro-inflammatory environment via the recruitment of macrophages that further drive tumorigenesis [20]. However, the regulatory mechanisms of SASP-induced activation of macrophages remain unclear.

Emerging data have revealed that the NF-κB signaling pathway is crucial and involves the regulation of the SASP [21, 22]. The activation of NF-κB is subject to a complex regulatory network, in which IkappaB (IκB) proteins constitute the most important family of factors in the activation of the NF-κB signal transduction pathway [23]. Additionally, B-cell leukemia 3 (Bcl3) has been identified as a member of the IκB family [24–26]. In contrast to other IκB proteins that bind to NF-κB in the cytoplasm, Bcl3 is primarily a nuclear protein and is involved in regulating the activity of NF-κB [27]. Bcl3 is closely related to some inflammatory diseases [24, 26, 28–34]. Furthermore, Bcl3 has been shown to be expressed in various hematopoietic and solid cancers [35–38]. However, to our knowledge, no study has yet determined whether Bcl3 might play a role in the SASP.

Therefore, we aimed to investigate the effects of SASP of hepatocytes on inflammatory microenvironment and hepatocarcinogenesis in a mouse model of HCC. In this study, we want to figure out how does senescent hepatocytes affect inflammatory microenvironment and whether hepatic SASP was involved. On the other hand, what mediates hepatic SASP and if Bcl3 mediates this process.

## Materials and methods

### Patients and tissue specimens

Specimens of HCC tissues were obtained from 74 HCC patients who underwent hepatic resection at the Shanghai Eastern Hepatobiliary Surgery Hospital from 1997 to 2007. These patients included 65 men and 9 women, with a median age of 41.59 years (range: 21–71), and all the specimens were subjected to IHC analysis. For the analysis, the IHC results were assigned a mean score, from 0 to 12. IHC staining index (values, 0–12) was determined by multiplying the staining intensity score with the positive area score. The intensity was scored as follows: 0, negative; 1, weak; 2, moderate; and 3, strong. The frequency of positive cells was defined as follows: 0, less than 5%; 1, 5% to 25%; 2, 26% to 50%; 3, 51% to 75%; and 4, greater than 75%. Each component was scored independently and summed for the results. For statistical analysis, scores of 0 to 7 were considered the low expression, and scores of 8 to 12 considered high expression. Prior informed consent was obtained, and the study protocol was approved by the Ethics Committee of the Shanghai Eastern Hepatobiliary Surgery Hospital.

### Animal models

#### DEN and CCl_4_-induced primary mice HCC model

Bcl_3_ knockout (*Bcl3*^*−/−*^) mice were obtained from Prof. Xiaoren Zhang [39]. These mice and C57BL/6 wild type (*WT*) were housed in a pathogen-free animal facility. After birth, randomly assign litters of male pups to treatment groups. Male mice are selected because male gender is a risk factor for human HCC. Mice received a single dose of diethylnitrosamine (DEN, Sigma, St. Louis, MO) (25 µg/g bw i.p.) at day 15 post-partum. Starting 2 weeks after DEN, mice followed by weekly injections of CCl_4_ (0.5 µl/g bw i.p., 1 injection/week) [40, 41]. Mice were sacrificed 48 h following the last CCl_4_ injection. Nodule number and size were documented as described by counting and measuring the diameter of each lesion using a caliper. The animal protocols were approved by the Second Military Medical University Animal Care Committee.

### Depletion of macrophages in vivo

Gadolinium chloride hexahydrate (GdCl_3_^.^6H_2_O; Sigma-Aldrich) (10 µg/g bw i.p., twice a week) was administered at 8 weeks after DEN injection; control group mice injected the same volume of normal saline twice a week.

### Histological examination

Liver samples were fixed in 4% paraformaldehyde, paraffin-embedded, and sectioned. HE, IHC staining, and immunofluorescence were performed. Each sample was independently assessed and scored by three pathologists, blinded to the study protocol.

### Immunohistochemistry (IHC) and immunofluorescence (IF)

IHC analysis was performed using the following antibodies: rabbit anti-p21 (diluted 1:100, Abcam, Cambridge, UK), anti-Bcl_3_ (diluted 1:100, Abcam, Cambridge, UK), anti-Bcl3 (diluted 1:100, Protechtein) anti-IL8 (diluted 1:100, Abcam, Cambridge, UK), anti-CD68 (diluted 1:100, Abcam, Cambridge, UK), anti-iNOS (diluted 1:100, Abcam, Cambridge, UK), and anti-CD163 (diluted 1:200, Abcam, Cambridge, UK). The detailed method has been published previously [42]. Five fields in each section were randomly selected to calculate the ratio of positive expression area. For the analysis, the positive intensity of IHC is calculated by counting the number of positive cells in the field of random collection for each marker. For IF, rabbit anti-HNF-4α (diluted 1:50, Abcam, Cambridge, UK), anti-p21 (diluted 1:50, Abcam, Cambridge, UK), anti-Bcl3 (diluted 1:50, Abcam, Cambridge, UK), and anti-IL8 (diluted 1:50, Abcam, Cambridge, UK) were used.

### SA-β-gal staining

SA-β-gal staining was performed using a senescence-associated β-galactosidase kit (Cell Signaling Technology, #9860). Frozen sections of mice liver samples were washed twice with PBS, fixed in 1× fixative for 15 min at room temperature, washed, and add the working solution of β-galactosidase plus X-Gal incubated overnight at 37 °C in a dry incubator (no CO2). The senescent cells were observed under an optical microscope (Olympus IX70) and counted from three random vision fields.

### RNA and cDNA preparation for standard qPCR and PCR array analyses

Total RNA was extracted using Trizol reagent according to the manufacturer’s protocol. Real-time PCR was performed in a total reaction volume of 20 µl (10 µl SYBR green (DBI-2220), 1 µl forward and reverse specific primers, respectively, 2 µl complementary DNA and 7 µl ddH_2_O). PCR conditions used were: 95 °C for 10 min, followed by 40 cycles of 95 °C for 15 s, 60 °C for 30 s, and 72 °C for 30 s. The relative amounts of qPCR products were determined with the ΔΔCt method to compare the relative expression of target genes and housekeeping genes. The expression of the gene encoding β-actin was used as an internal control. Primer sequences were indicated in Additional file 1: Table S1. PCR array was performed using Customer-Specific PCR Array (Qiagen) mice liver tissue.

### Western blot

Protein samples were collected, and equivalent aliquots of protein were electrophoresed on a 10% sodium dodecyl sulfate/polyacrylamide gel in 1 × Tris–glycine buffer transfer to nitrocellulose membranes and incubated with primary antibodies, overnight at 4 °C. After that, the nitrocellulose membranes were incubated with secondary antibody for 1 h, at room temperature, mouse or rabbit human antibodies (Bioworld, diluted 1:5000). The membranes were exposed using an enhanced chemiluminescence reagent (Chemicon International, USA), and densitometric analysis with the Image J software was performed. The primary antibodies used in our experiment included p21 (Abcam, diluted 1:1000), Bcl_3_ (Abcam, diluted 1:1000).

### Statistical analysis

All experiments were performed at least three times. Analysis of variance was performed using GraphPad Prism 8.0 (GraphPad Software). Quantitative data were expressed as mean ± SD for each experiment. Significance between groups was performed using Student’s *t*-test. Heatmaps were generated using the R language package. Clinical data analysis was performed using SPSS 22.0. Kaplan–Meier and log-rank analysis were performed to compare patient survival between subgroups. Statistical significances are indicated by *p < 0.05, **p < 0.01, ***p < 0.001, and ****p < 0.001.

## Results

### The hepatic senescence-associated secretory phenotype increases during hepatocarcinogenesis in mice

**Fig. 1:**
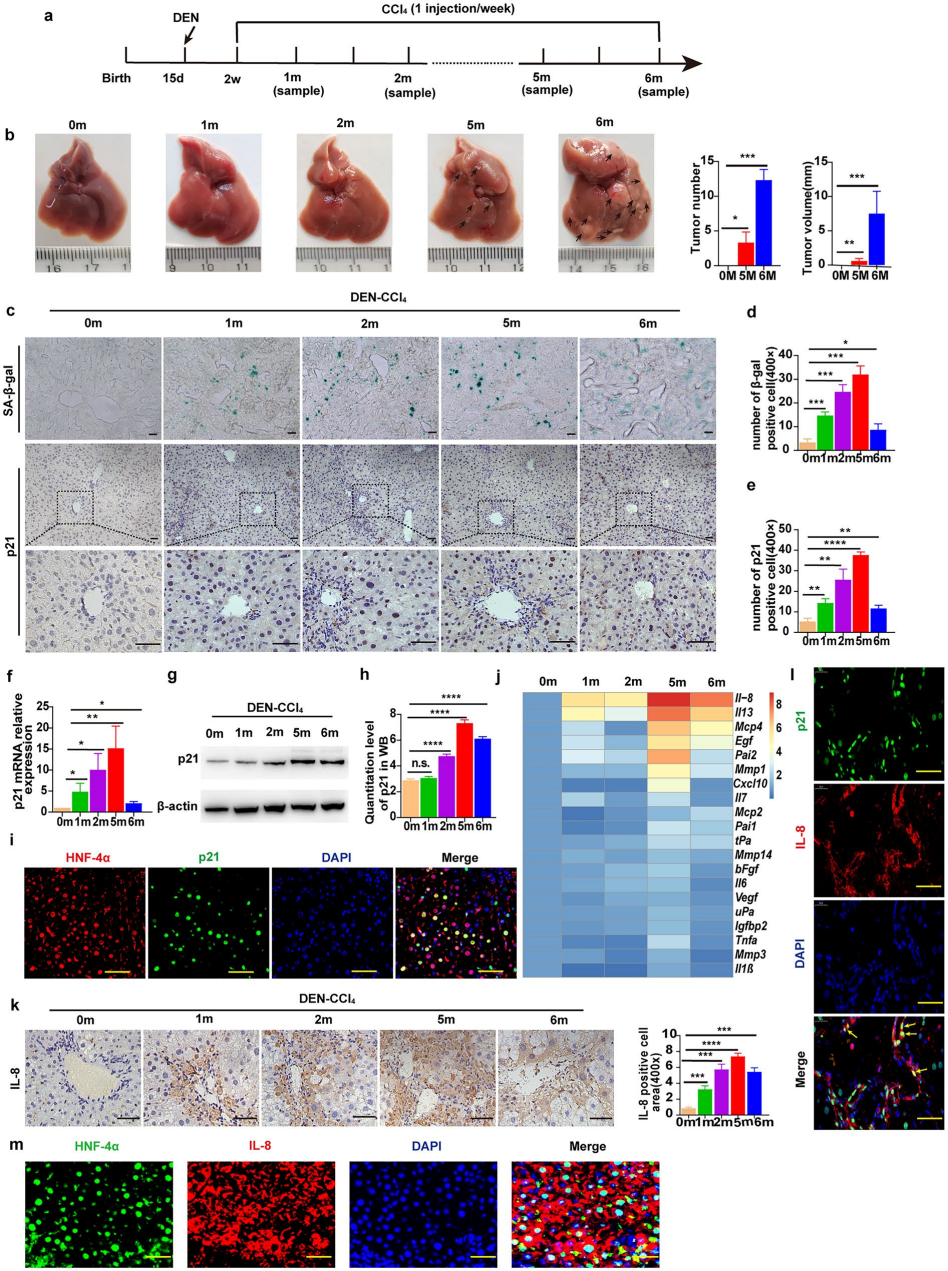
The Hepatic senescence-associated secretory phenotype is evident during hepatocarcinogenesis in mice. **a** Schematic representation of DEN-CCl_4_-induced HCC mouse model. **b** Representative liver morphology and the number of tumor nodules in the indicated groups. **c** Representative image of SA-β-Gal activity (blue) in frozen liver sections and IHC stained p21 in liver sections of the indicated groups. Site of zoomed image (below). Bar: 10 μm or 100 μm. **d**, **e** The expression of SA-β-gal (**d**) and number of p21-positive cells (**e**) were evaluated in the indicated groups. **f** Relative p21 mRNA levels were examined by qRT-PCR and normalized to the level in the control group. **g**, **h** p21 protein expression was analyzed by Western blotting. β-actin was employed as the loading control. **i** IF costaining of HNF-4α^−^positive cells (red) and p21-positive cells (green). Nuclei stained with DAPI (blue). Bar, 100 μm. **j** Heat maps of the typical SASP factor expression profile as determined using the R language package. **k** IHC staining and quantitation of IL-8 at different stages. Bar, 100 μm. **l** IF costaining of p21^−^positive cells (green) and IL-8^−^positive cells (red). Nuclei stained with DAPI (blue). Bar, 100 μm. **m** IF costaining of HNF-4α (green) and IL-8^−^positive cells (red). Nuclei stained with DAPI (blue). Bar, 100 μm. The data are presented as the mean ± SD. n = 5 for each group. *p < 0.05, **p < 0.01, ***p < 0.001, and ****p < 0.0001 between the indicated groups

We explored the role of senescent hepatocytes in hepatocarcinogenesis using the well-established N-nitrosodiethylamine (DEN)- and carbon tetrachloride (CCl_4_) (DEN-CCl_4_)-induced HCC mouse model (Fig. 1a, b). We found that the number of senescent hepatocytes increased during the induction of HCC. The results of senescence-associated beta-galactosidase (SA-β-gal) and immunohistochemical (IHC) staining of p21 was higher in the liver of the DEN-CCl_4_-treated mice than in the control group mice, specifically at 5 months (Fig. 1c–e). Moreover, consistent with these data, the mRNA and protein levels of p21 were significantly expressed 5 months after HCC induction (Fig. 1f–h) (Additional file 1: Table S1). These results indicate that cellular senescence plays an essential role in carcinogenesis. Nevertheless, recent studies have shown that the SASP in senescent stromal cells is closely related to tumorigenesis. However, hepatic cells are the primary cells through which liver functions are realized. Therefore, we costained p21 and the hepatocyte marker hepatocyte nuclear factor 4α (HNF-4α) in liver sections. We found p21-positive senescent cells (SCs) colabeled with HNF-4α (Fig. 1i), indicating that these SCs might mainly be derived from hepatocytes. Furthermore, recent findings have shown that SCs can secrete a variety of inflammatory cytokines, chemokines, and proteases, which are collectively referred as the SASP. Interestingly, our results revealed that senescent hepatocytes highly expressed multiple SASP factors, including but not limited to IL-8, after treatment with DEN-CCl_4_ (Fig. 1j). Thereafter, IHC staining showed that the IL-8 level was higher than it was in the control group, specifically at 5 months after HCC induction (Fig. 1k). Ultimately, we found p21^+^ SCs colabeled with IL-8 (Fig. 1l). In addition, IL-8 also co-localized with HNF-4α (Fig. 1m), indicating that senescent hepatocytes might secrete IL-8. These results suggested that hepatocyte senescence and the hepatic SASP correlated with hepatocarcinogenesis.

### Bcl3 expression is increased during hepatocyte senescence

**Fig. 2:**
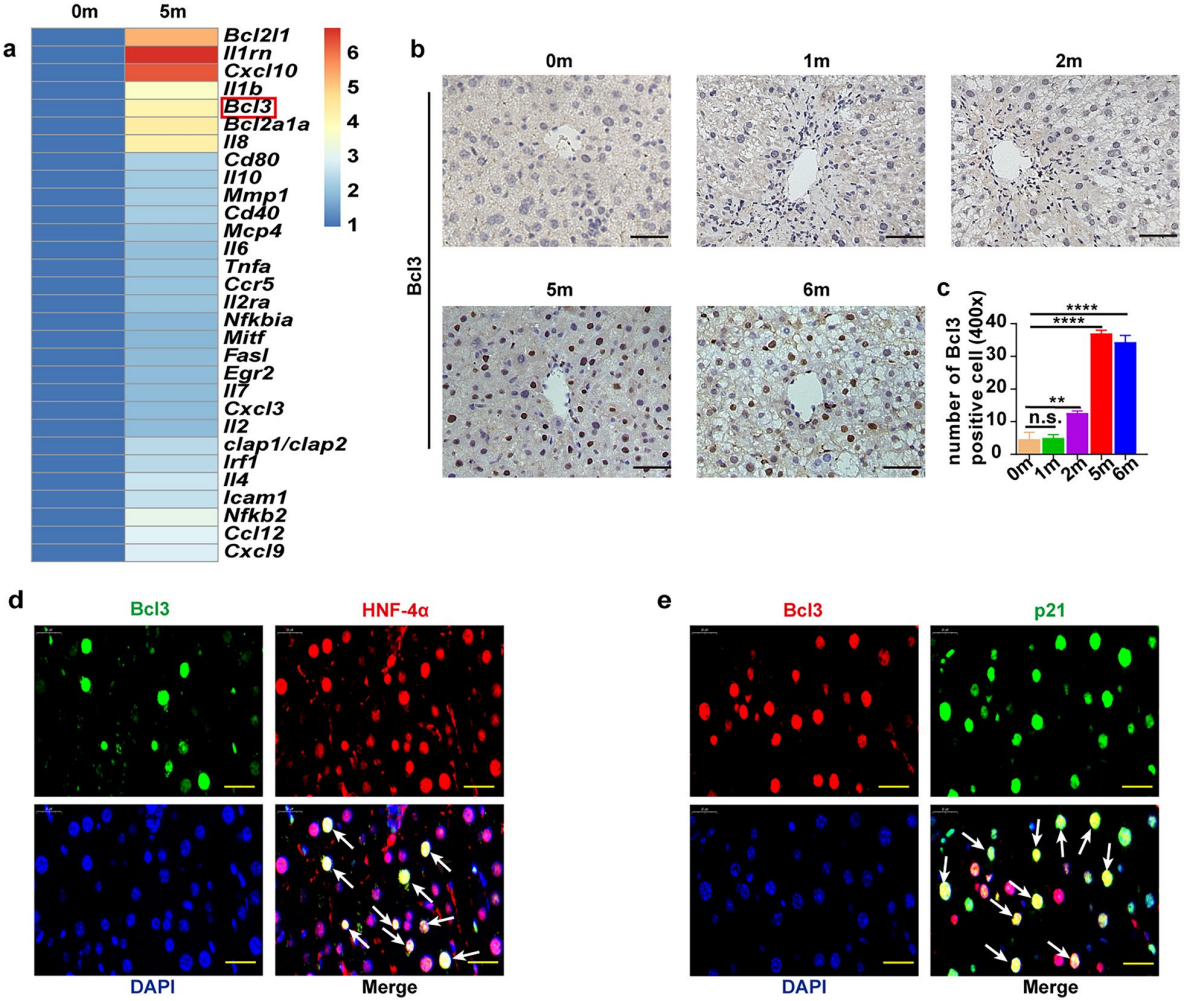
Bcl3 expression is increased during hepatocyte senescence. **a** Heat map of the 30 most significantly upregulated SASP or NF-κB genes in each group. **b**, **c** The expression of Bcl3 in each group was detected by IHC analysis. Bar, 100 μm. **p < 0.01 and ****p < 0.0001, as determined by t-test. **d** Representative image of IF costained Bcl3-positive cells (green) and HNF-4α-positive cells (red), and nuclei were stained with DAPI (blue) 5 months after HCC induction. Bar, 100 μm. **e** Representative image of IF stained Bcl3 (red) and p21 (green) 5 months after HCC induction. Bar, 100 μm

As we described previously, the expression of several canonical SASP factors was significantly increased 5 months after HCC induction compared with the control group. Emerging data have revealed that the NF-κB signaling pathway is the major pathway that stimulates the production of SASP factors. Thus, we selected DEN-CCl_4_-treated mice 5 months after induction and control group mice to investigate the correlation between the expression of the SASP and the NF-κB signaling pathway through PCR chip technology. We found that B-cell leukemia-3 (Bcl3), a nuclear member of the IκB (inhibitor of NF-κB) family, was significantly elevated. (Fig. 2a). Then, we performed IHC analysis of Bcl3 at different time points during the induction of HCC, and the results showed that Bcl3 was expressed consistency with previous observations (Fig. 2b, c). To confirm that Bcl3 was derived from senescent hepatocytes, a colocalization assay was performed. Specifically, we costained Bcl3 and HNF-4α, as well as Bcl3 and p21, and found through IF that Bcl3 was not only coexpressed with the hepatocyte marker HNF-4α but was also colocalized with the senescent marker p21 (Fig. 2d, e). These results strongly suggested that Bcl3 was derived from senescent hepatocytes. Altogether, our results indicated that Bcl3 expression was elevated during hepatocyte senescence.

### The hepatocyte SASP induces the activation of macrophages that participate in hepatocarcinogenesis

**Fig. 3:**
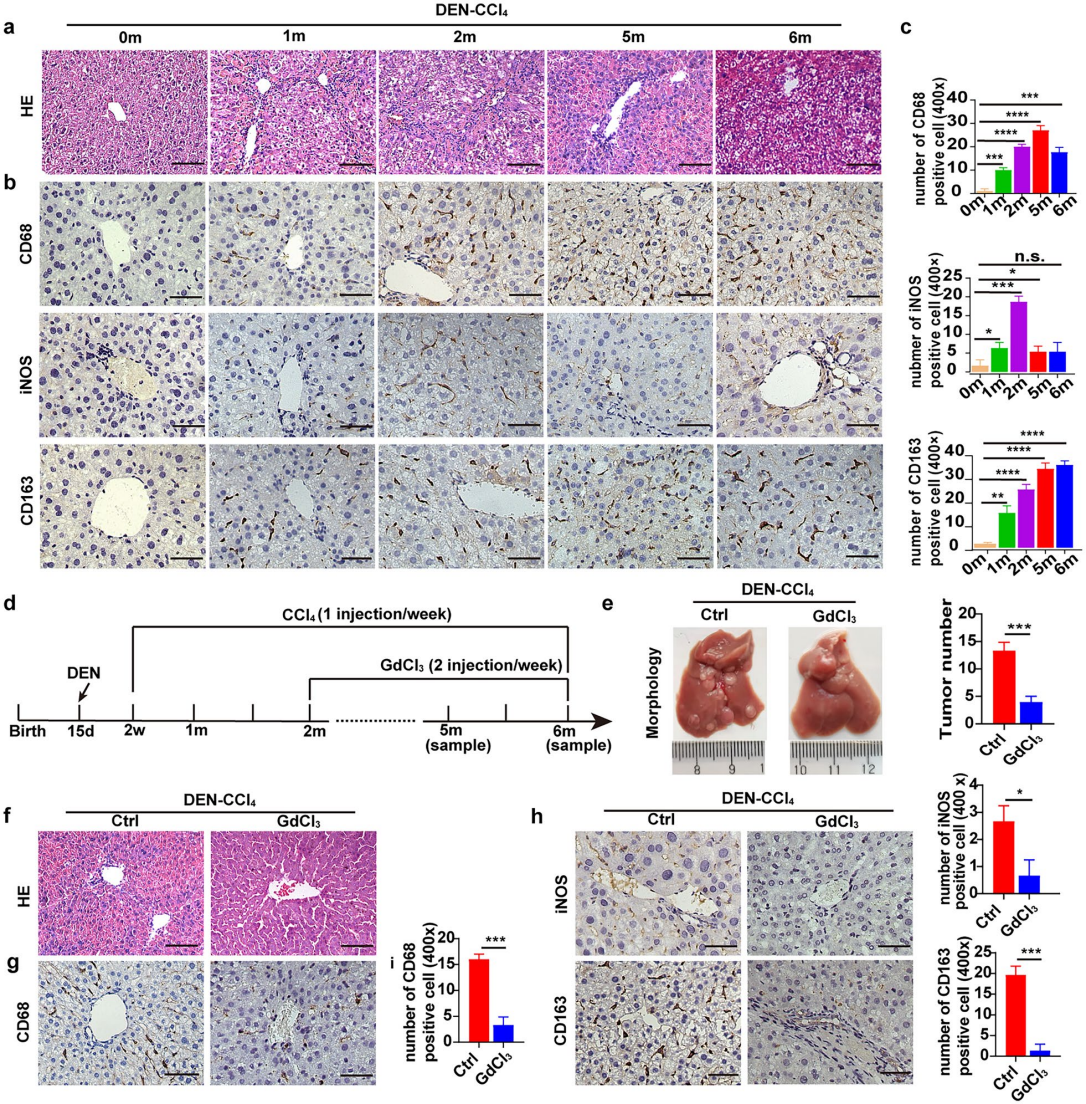
The hepatocyte SASP induces activation of macrophages that participate in hepatocarcinogenesis. **a** Representative image of HE stained liver in the indicated groups. Bars, 100 µm. **b**, **c** IHC staining and relative quantification of CD68, iNOS, and CD163 in liver tissues at different time points. Bars, 100 µm. **d** Schematic diagram of macrophage depletion by GdCl_3_ in DEN-CCl_4_-treated mice. **e** Representative image showing liver morphology of different groups at month 6, and the number of tumor nodules per liver was determined. **f** Representative imaging of HE-stained tissues in the indicated groups 5 months after HCC induction. Bars, 100 µm. **g**–**i** IHC staining and relative quantification of CD68, iNOS, and CD163 in the liver tissues in the indicated groups at 5 months. Bars, 100 µm. n = 5 for each group. *p < 0.05, **p < 0.01, ***p < 0.001, and ****p < 0.0001

Senescent cells can secrete a plethora of factors, including cytokines, chemokines, growth factors, and proteases, collectively termed the senescence-associated secretory phenotype (SASP). Notably, the SASP can activate immune cells and promote tumorigenesis. Therefore, we sought to investigate whether a similar response can be observed in senescent hepatocytes. Interestingly, we found that the expression of the total macrophage marker CD68 was increased 1 month after HCC induction and remained elevated for as long as 6 months during hepatocarcinogenesis. Consistent with these data, the expression of the M2 macrophage marker CD163 increased gradually during hepatocarcinogenesis. In contrast, the expression of iNOS, a marker of M1 macrophages, was maximal 2 months after HCC induction and then was decreased in the following months (Fig. 3b, c). These results indicated that macrophages can be activated during hepatocyte senescence. Therefore, we further explored the influence of macrophages on the occurrence of HCC. We depleted macrophages by intraperitoneal injection of GdCl_3_ (10 µg/g) twice weekly in a DEN-CCl_4_-induced mouse HCC model [43] (Fig. 3d). We found that tumorigenesis was dramatically suppressed after the macrophages were eliminated, implying that macrophage infiltration facilitated hepatocarcinogenesis (Fig. 3e). Furthermore, the liver tissue in the control group showed inflammatory cytokine production and infiltration of macrophages (CD68^+^ and CD163^+^), particularly at the portal regions, whereas inflammatory factor production and macrophage infiltration were significantly attenuated after macrophage depletion (Fig. 3f–i). Altogether, the hepatocyte SASP might induce the activation of macrophages, which then participate in hepatocarcinogenesis.

### Bcl3 knockout suppresses hepatocarcinogenesis by inhibiting the hepatic SASP and macrophage activation

**Fig. 4:**
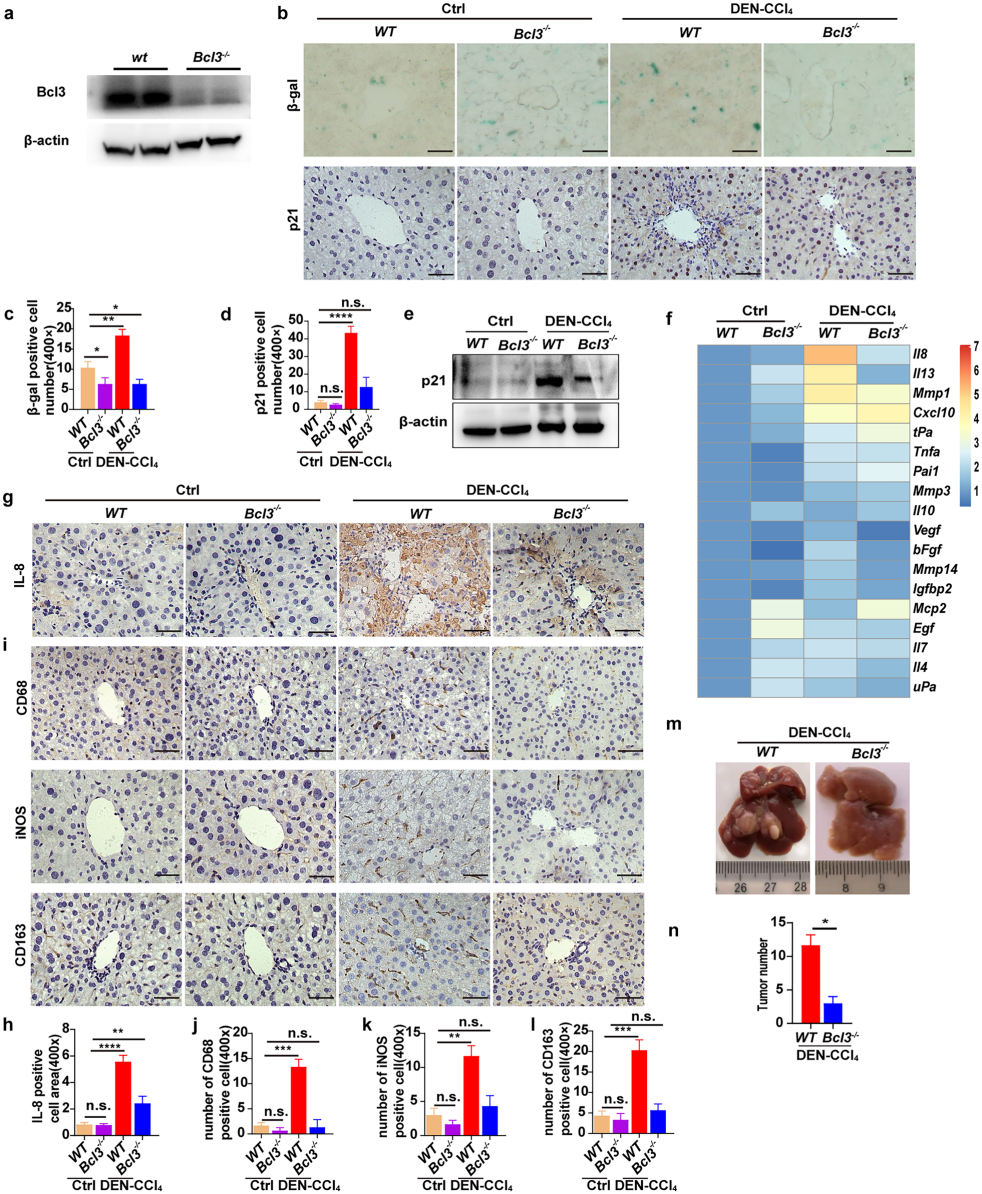
Bcl3 knockout suppressed hepatocarcinogenesis by inhibiting hepatic SASP factor production and macrophage activation. **a** The result of Bcl3 protein expression was analyzed by Western blotting. β-actin was employed as the loading control. **b** Representative images of stained SA-β-gal (blue) and IHC-stained p21-positive cells in the indicated groups. Bar, 100 μm. **c**, **d** Representative quantification of the number of SA-β-gal- (**c**) and p21-positive cells (**d**). **e** Western blotting was performed to analyze the protein expression levels of p21 in the indicated groups. **f** Heat map showing the expression of canonical SASP factors in the livers of *WT* and *Bcl3*^*−/−*^ mice in the indicated groups. **g**–**l** IHC staining and relative quantification of IL-8 (**g**, **h**) and CD68, iNOS, and CD163 (**i**–**l**) in the livers of *WT* and *Bcl3*^*−/−*^ mice in the indicated groups. Bar, 100 μm. **m**, **n** Representative gross photographs of the liver (**m**) and the number of tumors (**n**) in *WT* and *Bcl3*^*−/−*^ mice treated with DEN-CCl_4_. n = 5 for each group. *p < 0.05, **p < 0.01, ***p < 0.001 and ****p < 0.0001, as determined by t-test

We further studied the impact of Bcl3 knockout on hepatocyte senescence, SASP factor production, and macrophage activation, as well as the effect of Bcl3 knockout on the occurrence of HCC. The efficiency of Bcl3 knockout was verified by WB as indicated (Fig. 4a). SA-β-gal staining was detected, and fewer SA-β-gal-positive cells were found in the livers of Bcl3-knockout mice (*Bcl3*^*−/−*^) than in the livers of wild-type (*WT*) mice. In addition, the expression of p21 was also decreased in *Bcl3*^*−/−*^ mice (Fig. 4b–e). Furthermore, *Bcl3*^*−/−*^ mice showed reduced the production of SASP factors (Fig. 4f–h) and exhibited relatively few positive macrophages during hepatocarcinogenesis, as indicated by CD68, iNOS, and CD163 level (Fig. 4i–l). Furthermore, *Bcl3*^*−/−*^ mice showed a lower tumor burden than *WT* mice within 6 months of HCC induction, as indicated by decreased numbers of surface tumors (Fig. 4m, n). Therefore, these results suggested that knocking out Bcl3 decreased the number of senescent hepatocytes, mount of SASP factors produced, and extent of macrophage activation, thereby inhibiting hepatocarcinogenesis in mice.

### Increased expression of SASP factors and Bcl3 predicts poor prognosis in HCC patients

**Fig. 5:**
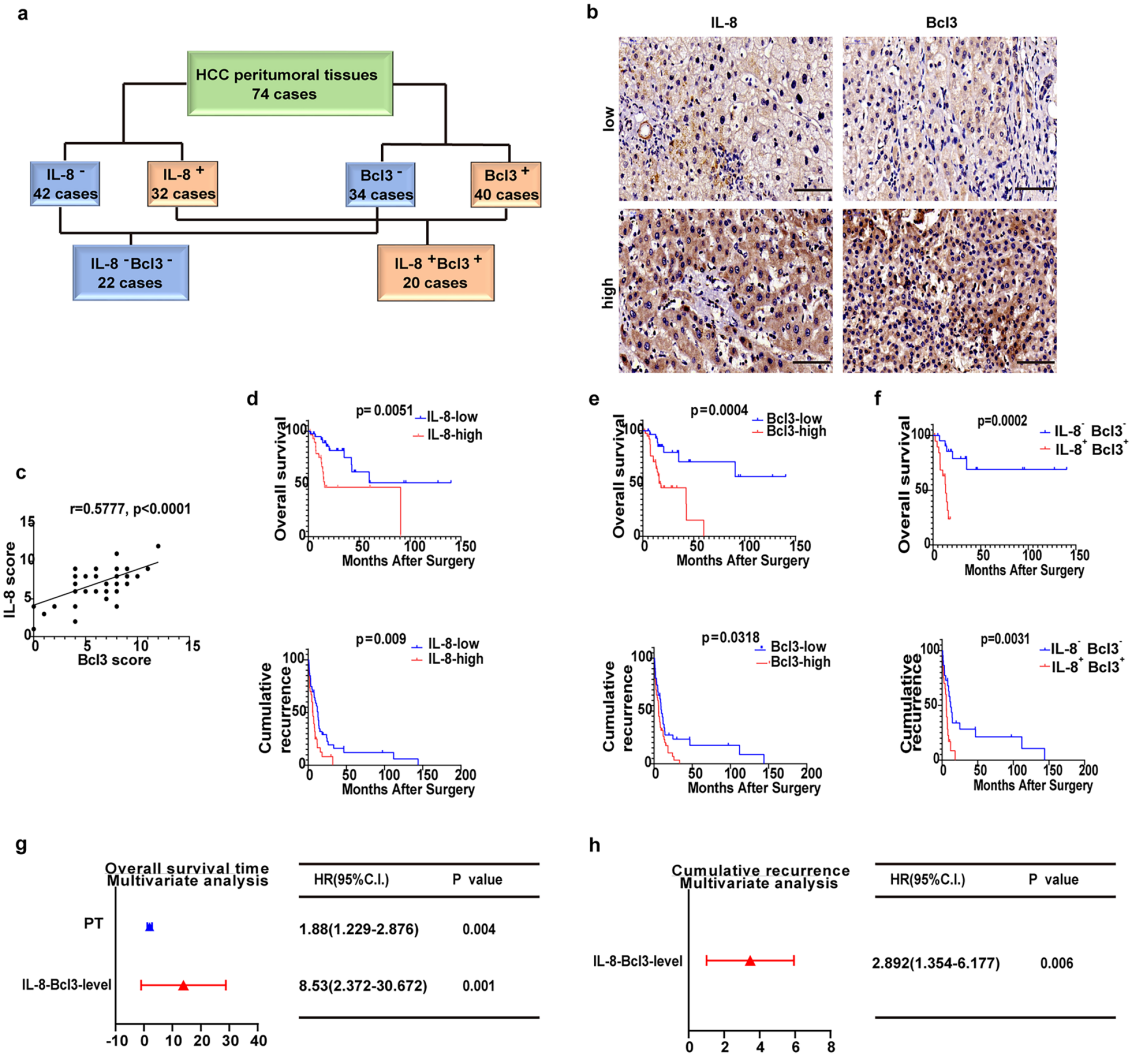
Increased SASP factor and Bcl3 expression predicted poor prognosis in HCC patients. **a** Schematic of the classification of 74 HCC peritumoral tissues. **b** IL-8 and Bcl3 expression was detected in HCC peritumoral tissues by IHC analysis. Bar, 100 μm. **c** Scatterplot with fitting line showing that the expression of Bcl3 strongly correlated with IL-8 expression. Pearson’s correlation analysis provided the correlation coefficient (r) and p-value. **d**–**f** Times of OS and DFS of patients after surgery were compared in each HCC group (Kaplan–Meier method), log-rank multiple comparison tests were performed to obtain p values. **g** Multivariate analysis of hazard ratios for the OS time of the 32 patients. **h** Multivariate analysis of hazard ratios for the DFS of the 32 patients. Abbreviations: C.I., confidence interval and HR, hazard ratio

Then, to validate the association between IL-8 and Bcl3 expression during hepatocarcinogenesis, we performed IHC staining with the SASP marker and Bcl3 in FFPE tissue specimens obtained from 74 HCC peritumoral tissues. According to the peritumoral IHC results, all 74 HCC-tissue-donating patients were categorized into the following groups: the IL-8 high expression (IL-8^+^) group (n = 34) and low expression (IL-8^−^) group (n = 40); the Bcl3 high expression (Bcl3^+^) group (n = 40) and low expression (Bcl3^−^) group (n = 34); IL-8^+^ Bcl3^+^ group (n = 22); and IL-8^−^Bcl3^−^ group (n = 20) (Fig. 5a, b). We analyzed the scores obtained by IHC scoring standards to examine the correlation between Bcl3 and IL-8 expression. Our results showed that IL-8 had a positive correlation with Bcl3 (r = 0.5777, p < 0.0001) (Fig. 5c). Furthermore, we compared the overall survival (OS) and disease-free survival (DFS) between patients with IL-8^+^ and IL-8^−^, and Bcl3^+^ and Bcl3^−^, and IL-8^+^ Bcl3^+^ and IL-8^−^Bcl3^−^ HCC cells on the basis of the Kaplan–Meier method and log-rank test, in which patients with IL-8^+^, Bcl3^+^, and IL-8^+^ Bcl3^+^ cells exhibited shorter OS times and earlier recurrence (Fig. 5d–f). Additionally, in the multivariate analyses, IL-8^+^ Bcl3^+^ was an independent prognostic factor for patient OS and DFS time (Fig. 5g, h). These data suggested that increased expression of the SASP and Bcl3 predicted poor prognosis in HCC patients.

## Discussion

An increasing number of studies have shown the importance and significance of hepatocyte senescence in chronic liver disease [6]. However, the specific role and mechanism of its secretory phenotype in the tissue microenvironment have not been fully elaborated. Studies have suggested that the SASP might play different roles according to cell type and microenvironment. For example, in mouse models, senescence of activated hepatic stellate cells reduced the secretion of extracellular matrix components and enhances immune-mediated clearance, thereby limiting liver fibrosis in mice [44]. However, another study has shown that obesity induced hepatic stellate cells to secrete various SASP factors and promoted hepatocarcinogenesis in mice [45]. Similarly, the hepatocyte SASP might have similar bidirectional effects [17]. Despite the rapid progress in the study of the SASP, there are still many aspects of the role and mechanism of the hepatocyte SASP in the development of liver cancer that are unknown. Therefore, exploring the regulatory role of the hepatocyte SASP in liver cancer provides an important theoretical bridge for an in-depth understanding of the pathogenesis of liver cancer and better diagnosis and treatment of liver cancer.

In this study, we chose the DEN-CCl_4_-induced HCC mouse model to study the effect of hepatic SASP factors on the microenvironment of hepatocarcinogenesis. We found that senescent hepatocytes significantly upregulated SASP factors, particularly IL-8, during hepatocarcinogenesis. We observed by immunofluorescence assay that IL-8 not only colocalized with senescent cells but also with hepatocytes; therefore, considering these results, we preliminarily thought that IL-8 might be secreted from senescent hepatocytes. In addition, the factors released by senescent hepatocytes activated macrophages and changed the tissue microenvironment, thereby promoting the development of liver cancer. This finding further suggested an important role for the hepatocyte SASP in the inflammatory microenvironment of hepatocarcinogenesis. The inflammatory microenvironment plays an important role in the development of tumors, especially in patients with liver cancer. Because the majority of patients with liver cancer in China have varying degrees of chronic hepatitis and liver injury, long-term chronic hepatitis or liver injury might cause hepatocyte necrosis, apoptosis or aging. Most previous studies have suggested that stromal cell senescence and fibroblast senescence are closely related to the formation of the tumor inflammatory microenvironment [16], but normal hepatocyte senescence is also related to tumorigenesis, and hepatocytes are the most important cells in liver cells. Furthermore, by analyzing the clinical tissue samples of 74 patients with hepatocellular carcinoma, we found that the expression of IL-8 in the tissue of para-cancer was closely related to the prognosis of patients with hepatocellular carcinoma. Therefore, our findings have important translational implications for the clinical treatment of patients with liver disease, as IL-8 antagonists might become a treatment option for patients with clinical liver disease [46, 47].

Based on the results, leading to the question: why does SASP factor expression gradually increase before hepatocarcinogenesis? We believe that with the extension of cancer induction time, the accumulation of injurious factors might lead to the continuous accumulation of senescent hepatocytes. Further, senescent hepatocytes secrete a variety of SASP factors and remodel the inflammatory microenvironment state before the formation of liver cancer. In this study, we focused on the most significant factor, IL-8, but perhaps other SASP factors also play important roles. IL-8, also known as CXCL8, is one of the most important components in the SASP. Several studies have shown that IL-8 is associated with the development of chronic hepatitis and liver cancer [8, 48–52]. However, the effect of IL-8 on hepatocarcinogenesis in the senescent microenvironment has not been addressed to date. IL-8 produced by senescent cells has been demonstrated to be associated with angiogenesis, proliferation of tumor cells and poor prognosis [53]. In addition, IL-8 is an effective leukocyte chemokine that can activate and recruit macrophages to senescent cells, where they exert their biological function [8]. Therefore, IL-8 derived from senescent hepatocytes provides some theoretical basis for targeted therapy of liver cancer. However, determining the functional role of IL-8 in liver diseases is challenging. The main limitations of the current study were the limited functional experimental methods used in animal models because the mice do not carry the IL-8-encoding gene; they express only gro, the gene product of which is the chemokine CXCL1/KC. However, mouse CXCL1 displays only ~ 68% sequence identity with human CXCL1. Given the differences between species, the roles of these chemokines are difficult to dissect in liver diseases [50, 54].

It is well known that the Bcl3 gene is a member of the IκB family. Bcl3 expression is elevated in a variety of tumors and is associated with tumor development and metastasis [35, 38, 55–57] and is closely related to inflammatory diseases [33, 58, 59]. However, there have been no reports on the relationship between Bcl3 and the hepatocyte SASP during hepatocarcinogenesis. First, we found that the expression of Bcl3 in normal mice roughly increased with prolonged cancer induction. Furthermore, we observed by immunofluorescence assay that Bcl3 colocalized with both hepatocytes and senescent cells, and knockout the Bcl3 gene in mice not only decreased the secretion of hepatocyte senescence-related inflammatory factors but also significantly inhibited hepatocarcinogenesis. Although, we used systemic Bcl3-knockout mice rather than liver-specific Bcl3-knockout mice, which somewhat limited our research. However, these findings still revealed that Bcl3 could affect the secretion of SASP-related factors in hepatocytes during hepatocarcinogenesis and provided an important theoretical basis for understanding the relationship between the hepatocellular SASP and hepatocarcinogenesis.

In further observations, we found that SASP-induced M1-type macrophages were mainly expressed in the first two months of hepatocarcinogenesis, whereas among the SASP-induced macrophage population, M2 macrophages were activated to a significant degree in the later stages of hepatocarcinogenesis. What causes the shift in macrophage phenotype during hepatocarcinogenesis? Various SASP factors secreted by senescent hepatocytes create highly dynamic changes, which might lead to continuous changes in the inflammatory microenvironment during hepatocarcinogenesis. Therefore, we speculate that macrophage shifting might be due to the highly dynamic changes characteristically induced by the SASP factors that drive changes in the microenvironment. In the early stage of hepatocarcinogenesis, liver injury is mild, and the innate immune system is first activated to repair the injured liver tissue by secreting various cytokines, thereby limiting the occurrence of tumors. However, with the aggravation of injury, the levels of inflammatory factors secreted by senescent hepatocytes continue to increase, causing changes, which in turn alter the state of the local microenvironment, initiate an adaptive immune response, and lead to an immunosuppressive effect, thereby promoting hepatocarcinogenesis. As early as 2002, Mantovani et al. described the heterogeneity of macrophages, describing the plasticity of monocytes and macrophages in response to exposure to the external microenvironment [60]. Although the clinical significance of macrophages in tumor development has been reported, the role of their heterogeneity in liver cancer development is poorly understood. Our results further enrich the knowledge on the role of SASP factors in macrophages and hepatocarcinogenesis and provide some theoretical basis for further research and treatment.

Notably, our data were based on the mouse model of DEN-CCl_4_-induced HCC, and the development of mouse tumors was triggered by the seeding of tumor cells throughout the entire liver. In contrast, human cancer develops spontaneously in chronically damaged livers. Despite these differences between human disease and the mouse model, both human and mouse senescent liver tissues exhibit a similar precancerous senescent hepatocyte-induced secretion of inflammatory factors, including but not limited to IL-8, which enhanced the activation of macrophages, thus promoting tumor progression.

In conclusion, compared with previous studies, our current work has some notable strengths. First of all, our study showed that hepatic SASP-activated macrophages depend on Bcl3, which contributes to establishing a tumor-promoting microenvironment and indicates a novel role for Bcl3 in senescent hepatocytes. Secondary, the deleterious effect of SASP-linked events has raised the possibility that therapeutics targeting SASP factors or senescent cells are promising alternatives for overcoming SASP-induced side effects. However, considering the beneficial effects of the SASP, including its contribution to wound healing and tissue repair [7], selectively inhibiting deleterious SASP factor expression rather than eradicating senescent cells per se appears to be a more sagacious approach to circumventing potential pitfalls, particularly in the current era of precision medicine. Although there are some exciting findings presented herein, there are still some limitations to this study. Firstly, although we confirmed that IL-8 and Bcl3 are closely correlated with the occurrence and development of HCC through related experiments, the specific signaling pathways remain unknown, and more experiments are still needed for further exploration. In addition, the experiments performed were in the experimental exploration stage, and there is still much to explore before the results can be used in the clinic for true transformation into treatments.

## Supplementary Information

**Additional file 1: Table S1. **PCR prime.

## Data Availability

Data will be provided upon request.

## Abbreviations

Bcl3: B cell leukemia 3
BSA: Bovine Serum Albumin
CCl_4_: Carbon tetrachloride
DEN: Diethylnitrosamine
DAB: Diaminobenzidine
DAPI: 4’,6’-Diamidino-2-pheny-lindole dihydrochloride
EDTA: Ethylene Diamine Tetraacetic Acid
FITC: Fluorescent isothiocyanate
GdCl_3_: Gadolinium chlorid
HCl: Hydrochloric acid
HRP: Horseradish peroxidase
H&E: Hematoxylin and Eosin
IHC: Immunohistochemical
IL: Interleukin
NaOH: Sodium hydroxide
IκB: Ikappa B
NF-κB: Nuclear transcription factor-kappa B
PBS: Phosphate buffered saline
PCR: Polymerase chain reaction
PAGE: Polyacrylamide gel electrophoresis
PVDF: Polyvinylidene fluoride
qRT-PCR: Real-time quantitative Polymerase chain reaction
RT: Reverse transcription
Rpm: Revolutions per minute
SDS: Sodium dodecyl sulfate
TBS: Tris-buffered Saline
TBST: Tris-buffered Saline Tween-20
Tris: Tris(hydroxymethyl)aminomethane
WB: Western Blot
β-gal: β-Galactosidase
